# Screening of Potential Drug Targets Based on the Genome-Scale Metabolic Network Model of *Vibrio parahaemolyticus*

**DOI:** 10.3390/cimb47070575

**Published:** 2025-07-21

**Authors:** Lingrui Zhang, Bin Wang, Ruiqi Zhang, Zhen He, Mingzhi Zhang, Tong Hao, Jinsheng Sun

**Affiliations:** Tianjin Key Laboratory of Animal and Plant Resistance/College of Life Sciences, Tianjin Normal University, Tianjin 300387, China; ztt11235813@163.com (L.Z.); wbkjc@tjnu.edu.cn (B.W.); 13623930869@163.com (R.Z.); 13236069290@163.com (Z.H.); 18404964001@163.com (M.Z.)

**Keywords:** *Vibrio parahaemolyticus*, genome-scale metabolic network model (GSMN), essential metabolites, drug target, systems biology

## Abstract

*Vibrio parahaemolyticus* is a pathogenic bacterium widely distributed in marine environments, posing significant threats to aquatic organisms and human health. The overuse and misuse of antibiotics has led to the development of multidrug- and pan-resistant *V. parahaemolyticus* strains. There is an urgent need for novel antibacterial therapies with innovative mechanisms of action. In this work, a genome-scale metabolic network model (GMSN) of *V. parahaemolyticus*, named VPA2061, was reconstructed to predict the metabolites that can be explored as potential drug targets for eliminating *V. parahaemolyticus* infections. The model comprises 2061 reactions and 1812 metabolites. Through essential metabolite analysis and pathogen–host association screening with VPA2061, 10 essential metabolites critical for the survival of *V. parahaemolyticus* were identified, which may serve as key candidates for developing new antimicrobial strategies. Additionally, 39 structural analogs were found for these essential metabolites. The molecular docking analysis of the essential metabolites and structural analogs further investigated the potential value of these metabolites for drug design. The GSMN reconstructed in this work provides a new tool for understanding the pathogenic mechanisms of *V. parahaemolyticus*. Furthermore, the analysis results regarding the essential metabolites hold profound implications for the development of novel antibacterial therapies for *V. parahaemolyticus*-related disease.

## 1. Introduction

*Vibrio parahaemolyticus* is an opportunistic pathogenic marine microorganism widely distributed in marine, estuarine, and coastal environments [1]. It infects a broad range of aquatic organisms, including fish, shrimp, and mollusks [2,3], and is recognized as one of the primary causative agents of acute hepatopancreatic necrosis disease, which has caused significant economic losses in shrimp aquaculture worldwide [4]. Moreover, *V. parahaemolyticus* is a major foodborne pathogen in humans, with outbreaks commonly linked to the consumption of raw or undercooked seafood, thereby posing substantial risks to public health [5]. Outbreaks of foodborne illness caused by this pathogen are frequently reported in coastal countries, particularly due to the consumption of contaminated seafood such as oysters and shrimp [6]. Therefore, drug development efforts against *V. parahaemolyticus* must consider both animal and human health contexts. At present, antibiotics remain the predominant means of treatment and disease control related to *V. parahaemolyticus*. However, the widespread and often indiscriminate use of antibiotics in aquaculture has led to the emergence of multidrug-resistant strains of *V. parahaemolyticus*, which complicates clinical management and undermines treatment efficacy [7,8]. Anti-microbial resistance (AMR) has become a major global health crisis. It causes nearly 5 million deaths annually and imposes an escalating burden on healthcare systems worldwide [9]. The presence of multi-antibiotic-resistant bacteria in seafood and aquatic environments exacerbates this issue [10]. Although alternative approaches such as vaccines, probiotics, phytotherapy, and nanomaterial-based strategies have been explored, they often face considerable challenges related to cost, scalability, and safety, limiting their widespread application [11,12,13]. These issues collectively underscore the critical need for the development of new therapeutic strategies that are both effective and environmentally sustainable.

Targeted drugs represent a key focus in drug development research. They are recognized for their high specificity and minimal environmental impact. In recent years, the genome-scale metabolic network model (GSMN) has emerged as a powerful systems biology approach for investigating the physiological features of pathogens’ cells and identifying potential drug targets [14,15]. It integrates genomic information, metabolic pathway data, and various layers of omics data with mature reconstruction methods and wide applications [16,17]. Cheng et al. predicted anti-SARS-CoV-2 targets based on the integrated analysis of 12 published in vitro and human patient gene expression datasets on SARS-CoV-2 infection with GSMNs [18]. The *Mycobacterium tuberculosis* GSMN-TB model has been instrumental in identifying the essential genes that are vital for in vitro growth and persistence within host cells. The model has successfully recovered a high proportion of previously validated drug targets, thereby reinforcing its predictive accuracy and reliability for therapeutic exploration [19]. Compared with gene-centric and reaction-centric approaches, metabolite-centric approaches based on GSMNs are preferred for target prediction of pathogens because metabolites exhibit higher structural similarity to drug ingredients [20]. Drugs structurally similar to the substrates of metabolic enzymes have been found to be 29.5 times more likely to bind to enzymes than randomly selected drugs [21]. This finding provides theoretical support for structure-based drug target screening and highlights the potential of GSMN in drug repurposing and target prediction. In *Klebsiella pneumoniae*, a genome-scale metabolic network-based screening approach identified KdsA, a crucial component of lipopolysaccharides in the bacterial outer membrane, as a key candidate target by simulating bacterial growth under conditions that closely mimic the host environment [22]. The drug targets of the multidrug-resistant pathogen *Acinetobacter baumannii* AYE were also developed with its GSMN based on a metabolite essentiality analysis [23]. These studies present the capability of GSMNs to simulate pathogen behavior under physiologically relevant conditions, enabling the rational prioritization of metabolic targets with minimal off-target effects in the host.

In this work, a high-precision GSMN model of *V. parahaemolyticus*, named VPA2061, was reconstructed based on genomic data from five distinct subtypes of *V. parahaemolyticus*. Using VPA2061, the potential drug targets were systematically predicted through essential metabolite analysis and pathogen–host association screening. Additionally, by identifying structural analogs of these metabolites, the candidate drug components were further proposed. The results of this study offer valuable insights into the development of targeted therapies against *V. parahaemolyticus*.

## 2. Materials and Method

### 2.1. The Experimental Design

**Figure 1:**
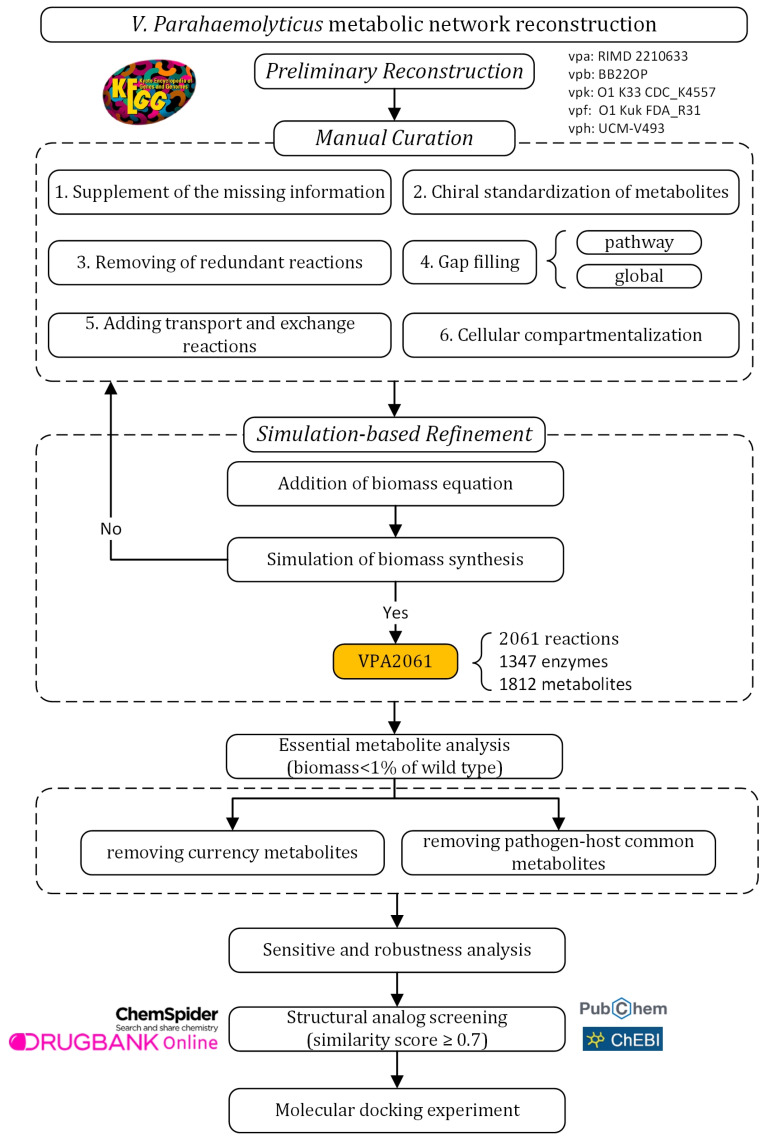
Experimental design of this work.

The metabolic network model was reconstructed following a standard workflow for reconstructing the GSMN [14,24]. The experimental design of this study is outlined in Figure 1. The reconstruction of the GSMN consists of three main stages: preliminary reconstruction, manual curation, and simulation-based refinement. In the preliminary reconstruction, metabolic data for five *V. parahaemolyticus* subtypes were retrieved from the Kyoto Encyclopedia of Genes and Genomes (KEGG) database [25]. The manual curation phase aimed to enhance the completeness and accuracy of the model. This included supplement of the missing information, chiral standardization of metabolites, removal of redundant reactions, performing gap filling at pathway and global levels, adding transport and exchange reactions, and cellular compartmentalization. Subsequently, simulation-based refinement was conducted to assess the capability of biomass synthesis. Additional biomass reactions were incorporated and simulation-based refinement was executed iteratively until the biomass synthesis was corrected simulated. The reconstructed VPA2061 was then used to analyze the essential metabolites, which were subsequently screened through removing currency metabolites and common pathogen–host metabolites to identify the potential drug targets. Finally, structural analog screening of the potential drug targets was performed using ChemSpider, PubChem, ChEBI, and DrugBank to explore potential compounds for drug design. The molecular docking experiment was conducted to evaluate the predicted structural analogs.

### 2.2. Reconstruction of the GSMN Model

#### 2.2.1. Preliminary Reconstruction

The metabolic data for the five *V. parahaemolyticus* subtypes used in the model reconstruction were retrieved from KEGG database. The data included genes, metabolic reactions (IDs, names, main reaction equations, full reaction equations, directionality), enzymes, metabolites (IDs, names), pathways, and subsystems for the five subtypes. These datasets were systematically organized and integrated to preliminarily reconstruct the GSMN for *V. parahaemolyticus*.

#### 2.2.2. Manual Refinement of the Model

##### Supplementing of the Missing Information

Missing information in the network, including main reactions, pathways, and subsystems, was supplemented to enhance the completeness of the model. Main reactions were added based on KEGG pathway maps and “RCLASS” data. Missing pathways were assigned according to the metabolites involved, and subsystem information was linked using pathway–subsystem relationships provided by the KEGG database. Furthermore, reactions that did not satisfy mass or charge balance were corrected by adding necessary reactants, such as H^+^ or H_2_O, to achieve proper stoichiometric balance.

##### Chiral Standardization of Metabolites

Metabolites with ambiguous chirality in the KEGG database were standardized to their biologically predominant forms. For example, all instances of D-Glucose (C00031) with unknown chirality were converted to alpha-D-Glucose (C00267), as the alpha form commonly existing in biological cells.

##### Removal of Redundant Reactions

To simplify the network and ensure accurate flux distribution, the redundant reactions were removed according to the following criteria: (1) Multi-step reactions: Retain the overall reaction if no branching occurred; otherwise, retain the stepwise reactions. (2) General reactions: Remove reactions involving classes of metabolites rather than specific metabolites. (3) Incomplete reactions: Remove reactions with undefined coefficients (e.g., “m” or “n”). (4) Macromolecular reactions: Remove reactions involving large molecules with R-group. (5) Duplicate reactions: Remove duplicated reactions resulting from the metabolite standardization.

##### Gap Filling

Gaps in the network affect the connectivity of the network. To address this issue, gaps in the network were filled at two levels: (1) pathway level: Reactions were added to connect weakly connected components (WCCs) within individual pathways [26]; (2) Global level: Reactions were incorporated to connect WCCs across the entire network. Suitable “gap-filling reactions” were identified from the KEGG database and integrated into the model to ensure comprehensive connectivity [27]. The selection of gap-filling reactions is primarily based on the annotations from the KEGG database. Gap-filling reactions are selected from the pool of all reactions in the KEGG database that are not included in the VPA2061 model. When multiple reactions are suitable for filling the same network gap, a pathway-prioritized screening approach is employed to reduce the false-positive additions. Specifically, reactions sharing the same pathway as the reactions flanking the gap are prioritized. If no such reaction with a matching pathway is found, reactions with smaller pathway numbers are prioritized, as smaller pathway numbers typically belong to the core metabolism. This method balances biological interpretability and network controllability, and it has been validated and applied in the reconstruction of various metabolic models [26,27].

##### Adding Transport and Exchange Reactions

Transport and exchange reactions were added based on the *Vibrio vulnificus* model VvuMBEL943, as *V. parahaemolyticus* and *V. vulnificus* share high protein similarity (75.6% match, E-value < 1 × 10^−5^). Specifically, 40.1% of proteins in *V. parahaemolyticus* and 75.6% of proteins in *V. vulnificus* exhibited similarity, indicating a high degree of similarity in nutrient uptake [28]. Therefore, the transport and exchange reactions from *V. vulnificus* were adopted as a reference. Exchange reactions were included to facilitate the uptake of extracellular metabolites (nutrients) and the secretion of waste products, while transport reactions enabled the movement of metabolites between intracellular and extracellular compartments. These reactions encompassed amino acids, DNA/RNA, phospholipids, small molecules, lipids, glycogen, and peptidoglycan to meet the metabolite exchange demands of *V. parahaemolyticus*.

##### Cellular Compartmentalization

The model was divided into two compartments: cytosol [c] and extracellular space [e]. Metabolites involved in metabolic reactions were assigned to the cytosol, while those involved in transport reactions were assigned to both compartments. Exchange reactions were assigned to the extracellular compartment. Metabolites in different compartments were treated as distinct entities.

#### 2.2.3. Simulation-Based Refinement

##### Addition of Biomass Equation

Biomass composition is typically determined based on experimental data on cellular nutrient content, which is essential for accurately representing the biosynthesis of major cellular components (e.g., amino acids and fatty acids) and predicting the growth rate of cells. Due to the lack of experimental data on the nutrient composition of *V. parahaemolyticus*, the biomass equation from the closely related *V. vulnificus* model, VvuMBEL943, was used to assist in constructing the biomass equation for *V. parahaemolyticus*.

##### Simulation of Biomass Synthesis

Simulations based on the GSMN were performed using the COBRA Toolbox v3.0 [29], a MATLAB (version R2021b)-based toolbox for metabolic network analysis. The reconstructed network was formatted according to COBRA requirements and imported into MATLAB for simulation. To calculate the cell growth rate, the biomass equation was set as the objective function, and Flux Balance Analysis (FBA) was performed.

The initial simulation result of the biomass synthesis rate is 0, indicating that the model could not synthesize biomass. To resolve this issue, the flux constraints of certain exchange metabolic reactions were manually adjusted to correct the difference in metabolite requirements between *V. vulnificus* and *V. parahaemolyticus*, until the model achieved a non-zero biomass synthesis rate, confirming its ability to simulate the growth of cells.

### 2.3. Essential Metabolite Analysis

Essential metabolite analysis was conducted by setting the fluxes of reactions associated with each metabolite to zero. For each metabolite, the fluxes of all its participating reactions were constrained to zero, and FBA was performed with the biomass setting as the objective function. If the maximum biomass synthesis rate calculated under these conditions was <1% wild-type biomass, the metabolite was deemed as essential; otherwise, it was classified as non-essential. All the metabolites in VPA2061 were tested to identify the essential metabolites of *V. parahaemolyticus*.

### 2.4. Sensitivity and Robustness Analysis

For the purpose of evaluating the sensitivity of essential metabolite predictions to biomass composition, ±50% perturbations were applied to each of the 8 biomass constituents (PROTEIN, DNA, RNA, PHOSPHOLIPID, CAV, LPS, PEPTIDO, GLYCOGEN) in the biomass equation, resulting in 16 distinct scenarios. The essential metabolites were predicted under each condition. In the biomass equation, the sum of coefficients for all biomass components (excluding ATP) is approximately 1. Therefore, when perturbing one component, compensatory adjustments were made to the other components to maintain this sum constant. The relative proportions among all non-perturbed components were preserved while applying an overall scaling factor to ensure their summed coefficients equaled or were close to 1.

In order to evaluate the robustness of reactions and essential metabolite predictions to biomass production, flux variability analysis (FVA) was performed using the COBRA Toolbox v3.0 [29]. The biomass reaction was set as the objective function, and the minimum and maximum allowable fluxes were calculated for all 2061 reactions in the VPA2061 model. The reactions with non-zero minimum and maximum fluxes and FluxRange < 15 were identified as the stable components under growth-optimized conditions. Although it is not a strictly rigid condition (i.e., FluxRange ≈ 0), these reactions consistently carry flux across multiple flux distributions compatible with maximum biomass and therefore represent non-redundant contributors under the modeled environment.

### 2.5. Screening for Structural Analogs for Essential Metabolites

The SMILES format chemical structures of all essential metabolites were obtained from the PubChem [30] (accessed on 4 June 2025), ChEBI [31] (accessed on 4 June 2025), and ChemSpider databases [32] (accessed on 4 June 2025) with the name and KEGG ID of the essential metabolites as the keywords. Subsequently the structural analogs of the essential metabolites were manually screened in the DrugBank database [33] (accessed on 4 June 2025) using Tanimoto coefficients based on 2D molecular fingerprints, with the SMILES format strings as the search terms. The structural similarity search function in DrugBank was employed to identify the compounds with high structural similarity to the essential metabolites. To accurately identify structural analogs of essential metabolites, only compounds with a Tanimoto similarity score ≥ 0.7 were considered as the structural analogs. This threshold has been widely applied in virtual screening studies to retain biologically meaningful structural similarity. For example, Chen et al. [34] adopted the same structural similarity criterion in their study of drug screening targeting the ABCA1 pathway. Similarly, Szilágyi et al. [35] noted in a large-scale drug network screening study that this threshold helps identify candidate molecules with functional similarity. All the structural alignments were performed in the standard drug library (approved, investigational, experimental) to ensure that the resulting structural analogs have potential pharmacological development value.

### 2.6. Molecular Docking Experiment

In order to assess the binding plausibility of the identified structural analogs, molecular docking simulations were conducted for representative metabolite–drug pairs. The essential metabolites were searched in the STITCH database [36] (accessed on 13 July 2025) using their names as the keywords to identify the interaction relationship between the essential metabolites and target proteins. The protein with the highest interaction score was selected as the target for subsequent molecular docking experiments. The structure analog exhibiting the highest similarity score was chosen as the ligand for the molecular docking experiment as well as the essential metabolites. Ligand structures were obtained from the DrugBank database (accessed on 13 July 2025), and the structures of target proteins were obtained from the Uniprot database [37] (accessed on 13 July 2025). Docking was performed using the SwissDock platform (www.swissdock.ch) [38] (accessed on 13 July 2025) based on the EADock DSS algorithm under default parameters. Ligand preparation, target pocket prediction, and docking run generation were all handled within the SwissDock interface. The resulting docking poses and binding energy predictions were compiled for further analysis.

## 3. Results

### 3.1. Preliminary Reconstruction and Manual Curation of the GSMN for V. parahaemolyticus

#### 3.1.1. Preliminary Reconstruction of the Model

The initial reconstruction was based on the data from five different subtypes of *V. parahaemolyticus* obtained from the KEGG database. The strain IDs and the number of KEGG Orthologues (KOs) for these subtypes are detailed in Table 1. KO numbers were used as a reference to align the metabolic data across the five subtypes. In total, there are 2390 KOs in the five subtypes after deduplication, in which 736 KOs were mapped to a consolidated metabolic reaction dataset for *V. parahaemolyticus* subtypes. The metabolic reactions of the five subtypes mapped with KOs were combined to obtain the preliminarily reconstructed metabolic network, which includes 736 KOs and 1664 reactions.

#### 3.1.2. Manual Curation of the Model

During the manual curation process, the preliminary reconstructed metabolic network was refined using relevant metabolic and annotation data from the KEGG database. In order to address the imbalances, 27 reactions with mass or charge discrepancies were corrected by adding appropriate metabolites or protons, ensuring that all reaction equations in the model achieved mass and charge balance. Additionally, main reaction information for 303 reactions, pathway information for 263 reactions, and subsystem information for 1140 reactions were supplemented based on the KEGG database. A total of 59 reactions were removed, including 3 incomplete reactions, 31 general reactions, and 28 macromolecular reactions. Also, 54 multi-step reactions were processed. Furthermore, the chirality of six metabolites in 115 reactions was standardized by setting the chiral ambiguous substances to the α-configuration, including glucose, fructose, galactose, glucose-6-phosphate, fructose-6-phosphate, and fructose-1,6-bisphosphate.

For the purpose of enhancing the connectivity of the network, gaps were filled at both the pathway and global scales. In total, 160 reactions were added, in which 117 were added at the pathway scale and 43 were added at the global scale. By performing the gap-filling step, the number of WCCs in the network was reduced from 318 to 102. The decrease in WCCs indicates that the gap-filling step significantly improves the connectivity of the network.

In order to obtain the nutrients from the environment for the growth of the cells, the transport and exchange reactions were added to the model. These reactions were added by referencing the GSMN of *Vibrio vulnificus* VvuMBEL943. Finally, 163 transport reactions and 123 exchange reactions were incorporated, encompassing transport and exchange reactions of 24 amino acids, 17 nucleotides, 9 carbohydrates, 6 vitamins and cofactors, 5 lipids, and 17 other small molecules.

After adding transport and exchange reactions, metabolites in the model were compartmentalized into intracellular and extracellular compartments. The model includes 1694 intracellular metabolites and 118 extracellular metabolites. Notably, when the same metabolite exists in both compartments, it is treated as two distinct metabolites in the simulation. The model includes 2061 reactions and 1812 metabolites in total after manual curation.

### 3.2. Simulation-Based Refinement of the Model

In order to enable the model to simulate the growth of *V. parahaemolyticus*, the biomass synthesis reactions should be incorporated into the network. These reactions were also adapted from *V. vulnificus* VvuMBEL943, including a biomass reaction and the synthesis reactions of nine precursor substances.

Since cross-species borrowing of metabolic information may introduce bias in model construction, a comprehensive demonstration was conducted from three perspectives: phylogeny, structural conservation, and metabolic capabilities. Firstly, according to the phylogenomic analysis by Ashok et al. [43], *V. parahaemolyticus* and *V. vulnificus* form neighboring monophyletic clades with 100% bootstrap support in a maximum-likelihood tree built from 2085 single-copy orthologous genes, supported by an average nucleotide identity (ANI) > 95% and in silico DNA-DNA hybridization (isDDH) > 70%. Secondly, genome-wide synteny analysis by Kim et al. [28] shows a high degree of conserved gene order between the large chromosomes of *V. parahaemolyticus* and *V. vulnificus*, suggesting a shared structural basis. Thirdly, in order to investigate the metabolic capabilities of the two species, all metabolic enzymes in VvuMBEL943 and VPA2061 were extracted, and the core metabolic enzymes involved in carbohydrate, amino acid, and nucleic acid metabolisms were screened. A comparative analysis of the metabolic enzymes and core metabolic enzymes between the two models was performed. The results show that 444 metabolic enzymes are shared in the two models, accounting for 88.27% (444/503) of the total metabolic enzymes in VvuMBEL943. Among these shared enzymes, 273 are core metabolic enzymes, representing 61.49% of the shared enzymes. These core metabolic enzymes constitute 79.36% (273/344) of the total core metabolic enzymes in VvuMBEL943. This indicates that the majority of metabolic pathways in *V. vulnificus* are conserved in *V. parahaemolyticus*, supporting the feasibility of using the *V. vulnificus* transfer and biomass equations for constructing the *V. parahaemolyticus* metabolic network model. Collectively, leveraging the biomass equation of VvuMBEL943 for constructing VPA2061 is feasible across all three dimensions: phylogeny, structural conservation, and metabolic capabilities.

As a result, the final model comprises 2061 reactions. With the biomass reaction set as the objective function, the biomass synthesis rate calculated with the model using FBA was initially 0, which means the model cannot correctly simulate the synthesis of biomass. This may due to the incompatibilities between *V. vulnificus* and *V. parahaemolyticus* as all the transport, biomass, and precursor synthesis reactions were imported directly from the *V. vulnificus* model. To resolve this issue, all the limited conditions of flux for the *V. vulnificus* imported reactions were checked and finally the limitations of 86 reactions were adjusted. The limitations of 85 exchange reactions involved in the transport of essential amino acids, sugars, lipids, and vitamins were set to [−5, 1000] instead of [−1000, 1000] or [0, 1000] in VvuMBEL943, while the limitations of the exchange reaction for sodium ion were set to [−1, 1000] instead of [−1000, 1000] in VvuMBEL943. The adjusted limitations of these reactions were determined according to the published works [44,45,46]. Typically, for most substances that can be absorbed into the cell as nutrients, an upper bound for the uptake rate must be specified. This rate is commonly set to −5 mmol·g^−1^h^−1^. For trace elements, a smaller upper uptake rate is defined, with −1 mmol·g^−1^h^−1^ being a commonly used value in other models. With these curations, the maximum biomass synthesis rate calculated with the model was 1.8182 mmol·gDW^−1^h^−1^, indicating that the model already has the ability to simulate biomass synthesis.

The final GSMN after refinement includes 2061 reactions and 1812 metabolites (Supplementary File S1). It was named VPA2061. The features of VPA2061 are shown in Table 2.

### 3.3. Essential Metabolite Analysis

Drug targets are usually substances that are critical to the survival of pathogens, so that affecting these metabolites can effectively eliminate pathogens. Therefore, to identify the potential drug targets for *V. parahaemolyticus*, essential metabolite analysis was conducted on all of the 1812 metabolites in VPA2061, and 68 essential metabolites were identified through model-based prediction. In these metabolites, the extracellular metabolites were deleted because they are actually the same metabolites as those in the cytosol location. In addition, the precursors comprising biomass were also removed, such as protein, DNA and RNA, because they are actually a general term for a class of substance instead of a certain metabolite. After excluding 13 extracellular metabolites and 10 precursors of biomass, a final set of 45 essential metabolites was obtained (Supplementary File S2). However, these essential metabolites need to be further screened as not all of the metabolites are suitable as drug targets.

The refinement process focused on organism specificity and potential impacts on the host. Metabolites that are not suitable to be drug targets were excluded, including the following: (1) currency metabolites: universal metabolites such as ATP and NADH, which are ubiquitous across organisms and involved in numerous metabolic pathways; (2) metabolites existing in the host: given that *V. parahaemolyticus* is a common pathogen primarily affecting host species such as fish and shrimp, it is critical to ensure that drug targets are specific to the pathogen to avoid adverse side effects for the host.

To systematically identify the currency metabolites, a list of currency metabolites was referenced from the *V. vulnificus* model. The currency metabolites were compared with the predicted essential metabolites of VPA2061 and finally revealed 14 currency metabolites, as detailed in Table 3. These metabolites were subsequently removed from the essential metabolites list.

Consequently, further pathogen–host association screening of the essential metabolite list was undertaken to exclude metabolites shared in both *V. parahaemolyticus* and its host species. In this study, Pacific white shrimp (*Litopenaeus vannamei*) was used as the host species. To minimize potential side effects of drug candidates to the host, the list of essential metabolites was compared with the 1737 metabolites in the *L. vannamei* metabolic network [45]. Essential metabolites that overlapped in the pathogen and host networks were removed from consideration. As a result, 21 homologous metabolites present in *L. vannamei* were removed. With the removal of currency metabolites and host homologous metabolites, the final list of the potential drug targets includes 10 metabolites.

### 3.4. Sensitivity and Robustness Analysis

The biomass sensitivity analysis was performed by perturbing the stoichiometric coefficients of eight biomass precursors (excluding ATP) by ±50% and reassessing essential metabolites using a relaxed threshold (biomass < 1% of wild-type flux). Across all 16 perturbation scenarios, the same 68 essential metabolites were consistently identified, indicating that the prediction is robust to biomass composition uncertainty. These results indicate that essentiality predictions are robust to both biomass composition variation and exchange-bound settings, supporting the reliability of the prediction of essential metabolites.

To evaluate whether the predicted essential metabolites are involved in stable, consistently active pathways under growth conditions, a robust analysis of the reactions in the model was conducted with FVA. In total, 32 highly robust reactions were ultimately identified. Among these, 10 reactions incorporated 9 out of the 10 predicted essential metabolites, achieving a coverage rate of 90%. This result demonstrates that the predicted essential metabolites actively participate in growth-related metabolic pathways, contributing to their stability. The result of FVA further reinforces confidence in the reliability of the prediction results. The FVA result of all the reactions in VPA2061 is provided in Supplementary File S3.

### 3.5. Structural Analog Analysis and Drug Development Prediction

The primary components of drugs often exhibit high structural similarity to their target molecules. Accordingly, the predicted candidate drug targets were used to further screen for the potential drug molecules. The structural analogs in the DrugBank database were analyzed for the 10 candidate drug targets to evaluate the possible drugs containing these analogs. As a result, all of the 10 candidate targets were found to have one or more structural analog with high similarity scores. In total, 39 structural analogs were identified after deduplication. A summary of the structural analog screening results is presented in Table 4, while detailed information is provided in Supplementary File S4.

Based on DrugBank classification, the 40 structural analogs were annotated with one or more status labels, including approved, investigational, withdrawn, experimental, and nutraceutical. These metabolites cover 6 approved drugs, 3 investigational drugs, 29 experimental drugs, and 1 withdrawn drug. These structural analogs include energy and redox coenzymes, amino acid analogs, nucleotide sugar/peptidoglycan precursors, isoprenoid diphosphates, and plant-derived polyphenols, as shown in Table 5. These results show that all of the 10 candidate targets have potential value for drug development.

### 3.6. Molecular Docking of Essential Metabolites and Their Structure Analogs

In order to assess the functional plausibility of the screened analogs, molecular docking simulations were performed for the representative metabolite–drug pairs using SwissDock. Interaction relationships for five essential metabolites and their target proteins were identified via searches in the STITCH database (Supplementary File S5). The target proteins for three of these metabolites were included in the VPA2061. These metabolites are ADP-L-glycero-D-manno-heptose, D-Alanine and UDP-N-acetylmuramate. For each metabolite, the interacting protein with the highest interaction score was selected as the target protein. The structural analog exhibiting the highest similarity score was selected as the ligand (Table 6). In terms of the molecular docking results, the conformation with the lowest binding free energy (most negative ΔG) was extracted for analysis. The results showed that the ATP binding to HldE yielded a binding free energy of −7.7 kcal/mol, approaching that of its native substrate ADP-L-glycero-D-manno-heptose (−8.2 kcal/mol). UDP-N-acetylmuramoyl-L-alanine binding to MurE yielded −8.2 kcal/mol, also close to its native substrate UDP-N-acetylmuramate (−9.1 kcal/mol). D-Alanine binding to HemE yielded approximately −5.1 kcal/mol, which was consistent with the expectations for a small molecule binding interface. These results indicate that the structural analogs are not only similar in structure but can also spatially mimic the binding mode of their native metabolites to the target proteins, suggesting potential competitive inhibitory functions.

## 4. Discussion

In this study, a GSMN of *V. parahaemolyticus* VPA2061 was reconstructed. The model was used to identify the potential drug targets with essential metabolites analysis. The essential metabolite analysis overcomes the limitations of traditional gene- or reaction-based target prediction methods. These conventional approaches often require the knockout of multiple genes to disrupt the pathogen’s robustness, given the presence of alternative metabolic pathways, potentially leading to complex off-target effects [40,41]. In contrast, essential metabolite analysis directly identifies metabolites critical for cell growth by simulating the removal of each metabolite in the metabolic network, thereby enabling the precise identification of potential drug targets. The sensitivity and robustness analysis further proved the reliability of this analysis method.

In the further screening of the predicted essential metabolites, filtering of currency metabolites and pathogen–host common metabolites was adopted. The host model for *L. vannamei* [45] represents the first published GSMN for shrimp. The model was reconstructed based on the genome and KEGG annotations and covers major metabolic pathways relevant to the physiology and nutrition requirements of *L. vannamei*. Since the sequencing and annotation of the *L. vannamei* genome are not perfect, it cannot be guaranteed that the model covered all the reactions of *L. vannamei*. This leads to the inevitable absence of some pathway information in the model. However, a conservative approach was still adopted to avoid false-negative results arising from model incompleteness. Therefore, in the pathogen–host association screening, only metabolites with confirmed annotations in the *L. vannamei* model were excluded from the target consideration.

Further structural analog analysis revealed that all of the 10 essential metabolites possess high structural resemblance to approved drugs, significantly enhancing the specificity and precision of essential metabolite analysis. Dobson et al. demonstrated that structural analogs are rational and practical starting points for drug development [47]. The results of molecular docking experiments further indicate that these structural analogs have potential competitive binding ability. In these structural analogs, two classes of drugs emerged as the most promising: ATP-related phosphonucleotides and gluconate-derived mineral salts. ATP functions as a central energy carrier and signaling molecule in shrimp. Studies have demonstrated that dietary supplementation with ATP analogs, such as 5-aminolevulinic acid, enhances intracellular ATP concentrations and upregulates immune-related gene expression, significantly enhancing shrimp survival under *V. parahaemolyticus* challenge [48]. These findings highlight the potential of ATP analogs as dual-function agents—disrupting bacterial metabolism while enhancing host immunity. In addition, several gluconate-based mineral supplements, including calcium gluconate, zinc gluconate, and copper gluconate, demonstrated high structural similarity to essential metabolites. These mineral compounds have already been incorporated into shrimp aquaculture as water-soluble additives or dietary supplements, contributing to ionic balance, stress resistance, and immune modulation [49,50]. Their established safety, availability, and bioactivity make them ideal candidates for repurposing as host-compatible metabolic inhibitors.

Moreover, several analogs mimic nucleotide sugars and peptidoglycan precursors, such as UDP-N-acetylmuramoyl derivatives, which are central to bacterial cell wall biosynthesis. These targets are conserved across Gram-negative pathogens and largely absent in eukaryotic systems, suggesting a high degree of specificity with minimal toxicity [51]. Additionally, natural compounds such as tannic acid, which also showed structural similarity, possess both antimicrobial and immunostimulatory effects and have been reported to improve shrimp resistance to bacterial infections [52].

The structural analogs of the predicted drug targets span a wide range of drugs, many of which are already in clinical use, including ATP, adenosine phosphate, NADH, alanine, D-alanine, serine, D-serine, tannic acid, calcium gluconate, zinc gluconate, and copper gluconate. This provides a solid foundation for the design of targeted therapies against *V. parahaemolyticus* by utilizing the pharmacological properties and mechanisms of these drugs. This approach would offer new avenues for developing innovative antimicrobial strategies.

Compared to previously published GSMNs such as VvuMBEL943 for *V. vulnificus*, the VPA2061 model offers distinct advantages in scope and applicability. It integrates genomic and metabolic data from five *V. parahaemolyticus* strains, including both clinical and environmental isolates, allowing for better representation of intra-species diversity and ecological flexibility. Moreover, *V. parahaemolyticus* poses a unique threat in aquaculture environments, where excessive antibiotic usage promotes the emergence and dissemination of antimicrobial-resistant strains. Aquaculture-associated resistance can cross ecological boundaries, affecting human, animal, and environmental health through shared water systems and food chains [9]. In this context, developing a strain-inclusive and ecologically relevant GSMN provides a necessary systems biology foundation for identifying robust, resistance-mitigating drug targets. VPA2061 provides a useful supplement to the existing *Vibrio* GSMN research in species selection, modeling strategy and practical application value.

Although this study identified the essential candidate metabolites and structurally similar drug-like compounds with potential antimicrobial relevance, all findings were obtained through in silico simulations. The core objective of this study is to identify potential drug targets through simulation methods, thereby providing theoretical support for subsequent experimental validation and functional screening, instead of direct therapeutic efficacy confirmation. Experimental validation, such as gene knockout studies, metabolic flux analysis, and in vitro antibacterial assays, is necessary to evaluate the biological relevance and therapeutic feasibility of these targets. In particular, the metabolic stability and universality of these candidate metabolites as drug targets under varying environmental conditions remain to be determined.

Additionally, due to the genetic diversity and serotype variability of *V. parahaemolyticus*, its metabolic characteristics may differ across strains or environmental conditions. Further studies should integrate epidemiological data to better understand the regulatory mechanisms and pathogenic roles of these metabolites in different strains.

Antibiotic resistance continues to pose a major challenge in the development of antimicrobial drugs. The essential metabolite-based screening approach provides a promising method for the creation of highly specific drugs with a lower risk of resistance. By targeting critical metabolic processes or using structural analogs for competitive inhibition, this approach allows for precise control of pathogen growth while minimizing the effects on non-target organisms. Importantly, this metabolite-based framework is not limited to *V. parahaemolyticus* and can be generalized to other bacterial pathogens with well-characterized metabolic networks.

## 5. Conclusions

In this study, a GSMN of *V. parahaemolyticus* named VPA2061 was reconstructed, which includes 2061 reactions and 1812 metabolites. Using this model, 10 key essential metabolites were identified through essential metabolite analysis and metabolite screening, providing potential drug targets for antimicrobial development. Additionally, by comparing structural analogs, 39 existing drugs structurally similar to the 10 essential metabolites were evaluated, several of which have demonstrated efficacy in competitive binding with the target proteins. The VPA2061 model provides a comprehensive framework for elucidating the metabolic landscape and pathogenic mechanisms of *V. parahaemolyticus.* More importantly, the identified essential metabolites and their structural analogs offer valuable starting points for the development of novel, targeted antimicrobial strategies, supporting more precise and effective disease management in aquaculture systems.

## Figures and Tables

**Table 1:** Data on five subtypes of *V. parahaemolyticus.*

Strain ID	Strain Name	Number of KOs	References
vpa	RIMD 2210633	2187	[39]
vpb	BB22OP	2197	[40]
vpk	O1 K33 CDC_K4557	2261	[41]
vpf	O1 Kuk FDA_R31	2275	[41]
vph	UCM-V493	2265	[42]

**Table 2:** Features of VPA2061.

Items	Number
Reaction	2061
Enzyme	1132
Metabolite	1812
Pathway	150
Subsystem	13

**Table 3:** List of currency metabolites.

Item	Name
C00002	ATP
C00003	NAD
C00005	NADPH
C00006	NADP
C00008	ADP
C00009	PI
C00010	CoA
C00014	NH3
C00015	UDP
C00016	FAD
C00041	ALA
C00022	Pyruvate
C00063	CTP
C00075	UTP
C00101	Tetrahydrofolate
C00105	UMP

**Table 4:** List of matched drugs.

KEGG ID	Name	Matched Structural Analogs
ADPHEP	ADP-L-glycero-D-manno-heptose	DB00171, DB00131, DB01992, DB00157, DB03147
C00133	D-Alanine	DB00160, DB01786, DB02952, DB00133, DB03929, DB19057, DB03320, DB04454
C00498	ADP-glucose	DB00171, DB00131, DB01992, DB00157, DB03147
C00692	Uridine-5’-Diphosphate-N-Acetylmuramoyl-L-Alanine-D-Glutamate	DB02314, DB01673, DB02196, DB03397, DB04147, DB03041, DB01861, DB02421, DB03501, DB01713, DB03723, DB03751, DB04097
C00993	D-Alanyl-D-alanine	DB07939, DB02518
C01050	UDP-N-acetylmuramate	DB01673, DB02196, DB03397, DB04174, DB02314, DB03041 DB01861, DB02421, DB03501, DB01713, DB03723, DB03751 DB04097, DB03161, DB02976, DB03488, DB02554, DB02485
C01212	UDP-N-acetylmuramoyl-L-alanine	DB01673, DB02196, DB03397, DB02314, DB04174, DB03041 DB01861, DB02421, DB03501, DB01713, DB03723, DB03751 DB04097, DB03161, DB02976, DB03488, DB02554
C04576	Pentagalloylglucose	DB03208, DB09372
C04877	UDP-N-acetylmuramoyl-L-alanyl-gamma-D-glutamyl-meso-2,6-diaminopimelate	DB02314, DB01673, DB04174, DB02196, DB03397, DB03041 DB01861, DB02421, DB03501, DB01713
C17556	di-trans,poly-cis-Undecaprenyl phosphate	DB07780, DB07841, DB02552

**Table 5:** Classification of structural analogs.

Category	DrugBank ID
Energy and Redox Coenzymes	DB00171, DB00131, DB01992, DB00157, DB03147
Amino Acid Analogs	DB00160, DB01786, DB00133, DB03929, DB19057, DB03320, DB04454, DB02518, DB07939, DB02952
Nucleotide Sugar/Peptidoglycan Precursors	DB02314, DB01673, DB02196, DB3397, DB04147, DB03041, DB01861, DB02421, DB03501, DB01713, DB03723, DB03751, DB04097, DB03161, DB02976, DB03488, DB02554, DB02485, DB04174
Isoprenoid Diphosphates	DB07780, DB07841, DB02552
Plant-Derived Polyphenols	DB03208, DB09372

**Table 6:** Results of molecular docking.

Essential Metabolite	Structure Analog	Target Protein	ΔG1 ^1^ (kcal/mol)	ΔG2 ^2^ (kcal/mol)
ADP-heptose	ATP	HldE	−8.2	−7.7
D-Alanine	D-Alanine	HemE	−5.1	−5.1
UDP-MurNAc	UDP-MurNAc-L-Ala	MurE	−9.1	−8.2

^1^ The most negative ΔG for the essential metabolite and its target protein; ^2^ the most negative ΔG for the structure analog and its target protein.

## Data Availability

All data generated or analyzed during this study are included in the Supplementary Files.

## Supplementary Materials

The following supporting information can be downloaded at: https://www.mdpi.com/article/10.3390/cimb47070575/s1.

## Abbreviations

GSMN	Genome-scale metabolic network model
KO	KEGG orthology
FBA	Flux balance analysis
WCC	Weakly connected component
FVA	Flux variability analysis
AMR	Anti-microbial resistance
ANI	Average nucleotide identity
isDDH	In silico DNA-DNA hybridization
